# Novel scoring system for predicting stone-free rate after flexible ureteroscopy lithotripsy

**DOI:** 10.1097/MD.0000000000040390

**Published:** 2024-11-01

**Authors:** Bin Yang, Shiwei Sun, Jinyao Wang, Jingyu Wang, Shuqin Liu, Yangbing Wei, Xiaofeng Yang

**Affiliations:** a Department of Urology, Third Hospital of Shanxi Medical University, Shanxi Bethune Hospital, Shanxi Academy of Medical Sciences, Tongji Shanxi Hospital, Taiyuan, China; b Department of Urology, Peking Union Medical College Hospital, Chinese Academy of Medical Sciences and Peking Union Medical College, Beijing, China; c Department of Urology, Taiyuan Central Hospital of Shanxi Medical University, Taiyuan, China; d Department of Radiology, Second Hospital of Shanxi Medical University, Taiyuan, China; e Department of Urology, First Hospital of Shanxi Medical University, Taiyuan, China.

**Keywords:** Flexible ureteroscopy, renal stones, stone/transverse process pixel ratio, stone-free rate, urolithiasis

## Abstract

This study aims to investigate the factors affecting the stone-free rate (SFR) of flexible ureteroscopy and laser lithotripsy (fURSL) for renal stones and establish predictive models by identifying their prognostic factors. We retrospectively examined 252 patients with renal stones who were treated with fURSL between July 2020 and April 2022. We analyzed the relationship between the patient’s clinical data (sex, age, and body mass index), stone status (side, size, location, stone/transverse process pixel ratio [STPR], and the CT value of stone [SCTV]), and SFR to determine the relevant factors and analyze their influence. Additionally, a nomogram was constructed based on these prediction results. A total of 252 patients were enrolled based on the inclusion and exclusion criteria. They were reviewed 7, 30, and 90 days postoperatively, with 46, 23, and 10 patients failing to discharge stones, respectively. Univariate Cox proportional hazard regression results revealed that the SFR was correlated with stone location, diameter (D1, diameter of stone measured by computed tomography [CT]; D2, diameter of stone measured by kidney-ureter-bladder abdominal radiography), SCTV, STPR, and operation time. Multivariate Cox proportional hazard regression was used to develop 2 predictive models for the SFR. The influencing factors of model 1 included D1, location, and SCTV, whereas those of model 2 were D2, location, and STPR. The results are shown in the nomogram. Receiver operating characteristic curves showed no significant difference between models 1 and 2 (*P* = .498), indicating that the nomogram was highly predictive. After 1000 resamples and internal self-validation, the C-indices of models 1 and 2 were 0.924 and 0.895, respectively, showing that the stone clearance predicted by the nomogram matched the actual situation. Stone location, size, and density (SCTV and STPR) were significant predictors of SFR after fURSL. The scoring system based on these factors may be used to guide optimal treatment strategy selection.

## 1. Introduction

Urolithiasis is one of the most common urological diseases worldwide. However, authoritative guidelines, including those approved by the European Association of Urology (EAU), American Urological Association (AUA), and Chinese Urological Association, consider that flexible ureterorenoscopy and laser lithotripsy (fURSL) are the indications of the renal stones whose diameter is no more than 20 millimeter (mm). Simultaneously, percutaneous nephrolithotomy (PCNL) is recommended for larger renal stones.^[1]^ Hyams et al^[2]^ conducted a multicenter follow-up study. They found that ureteroscopic laser lithotripsy (URSL) in 1 or multiple stages was effective in treating renal stones with a diameter between 20 and 30 mm, according to surgical experience and patient factors. In addition, Aboumarzouk et al^[3]^ proposed a systematic review and meta-analysis of fURSL for renal stones with diameters >20 mm. The results showed that fURSL could be a compelling alternative to PCNL in experienced centers. Furthermore, with the advancement of equipment for flexible ureteroscopy (fURS) and techniques for holmium laser, particularly the emergence of disposable fURS, and patients’ willingness to avoid the risk of severe complications of PCNL, fURSL is increasingly applied in the treatment of kidney stones.

However, many factors, other than the size of the stones, may influence the stone-free rate (SFR) of fURS; in turn, this may affect surgical outcome and patient satisfaction. Therefore, it is imperative for surgeons to select patients to undergo fURS, assess the surgical difficulty, and predict the surgical effect preoperatively. Several scoring systems for predicting the SFR of fURS have been proposed in the past few years,^[4–6]^ including the Resorlu–Unsal Stone score, Modified Seoul National University Renal Stone Complexity score, Tallness, Occupied lesion, Houndsfield unit evaluation score, and retrograde intrarenal surgery scoring system score. Common to all types of scoring systems is that many parameters are obtained based on computed tomography (CT). However, there are few reports on scoring systems based on other radiological methods. These scoring systems have a certain application value, though some shortcomings still require overcoming. It is necessary to establish a comprehensive, convenient, and stable scoring system.

Noncontrast CT (NCCT) is the preferred approach for diagnosing renal stones, as recommended by the EAU, AUA, and Chinese Urological Association guidelines. Mitterberger et al^[7]^ reported that kidney-ureter-bladder abdominal radiography (KUB) combined with ultrasound (US) for diagnosing renal stones was effective, with an accuracy, sensitivity, and specificity of 95%, 96%, and 91%, respectively; it could serve as an alternative to NCCT. KUB remains an important and popular technique in renal stone imaging. The sensitivity and specificity of KUB are estimated to be 57% and 76%, respectively.^[8]^ KUB can evaluate the stones’ density and roughly confirm location, size, and shape, and emits fewer radiations than CT.

Currently, there are few studies which have included both NCCT and KUB for predicting SFR after fURSL. Therefore, based on the reasons above, our center reviewed cases of renal stones treated with fURSL recently to predict the surgical effect of fURSL, and attempted to establish a prediction model for predicting SFR after fURSL using NCCT and KUB.

## 2. Methods

### 2.1. Study objects

Overall, 301 cases of renal stones treated with fURSL in Shanxi Bethune Hospital from July 2020 to April 2022 were retrospectively examined by KUB and NCCT before surgery and standard preoperative examination (including complete blood count, urinalysis, urine bacterial culture, serum creatinine, and estimated glomerular filtration rate, among others). The inclusion criteria were as follows: single renal stone, radiopaque stones on X-ray, no preoperative treatment, such as extracorporeal shock wave lithotripsy (ESWL) or medical expulsive therapy, and contraindications to PCNL or willingness of the patient to be treated with fURSL. The exclusion criteria were as follows: bilateral renal stones, musculoskeletal or renal malformation, pelviureteric tumor, transparent stones on X-ray, chronic kidney disease (stage 3 or above), and insufficient radiological examination data.

### 2.2. Methods

Preoperatively, all patients were treated with antibiotics. Additionally, patients with abnormal urine bacterial cultures were treated with antibiotics that were sensitive to the bacteria until the urine bacterial culture returned to normal. All operations were performed using fURS (zebra R, XFGC-FU-R, Bengbu City, China) and a holmium laser lithotripsy system (Lumenis Ltd, Irvine, California) with general anesthesia or combined spinal-epidural anesthesia. Furthermore, the operations were performed by 2 urologists with extensive experience in fURSL, and all patients were treated with a ureteral sheath (NavigatorHD, Boston Scientific, Natick, Massachusetts). Holmium laser lithotripsy with fragmentation and dusting was used. The energy level and frequency ranges of holmium laser to lithotripsy were 0.6 to 1.2 joule and 5 to 10 hertz, respectively. A stone extraction basket (NGage Nitinol Stone Extractor Modified Basket, COOK 1.7Fr × 115 cm) was used to remove the stone and grab as many large stone fragments as possible. After the surgery, the ureteral stents were indwelled for all patients and were removed 1 month postoperatively.

KUB and US were performed at 7 days, 1 month, and 3 months postoperatively to evaluate the SFR. In addition, if the patient was severely symptomatic or had abnormal vital signs, complete blood count, urinalysis, US, KUB, NCCT, and other examinations were performed to evaluate perioperative complications. The stone-free (SF) state was regarded as no detectable stone on the KUB, including stones with fragments <2 mm.

We compared the SF and non-SF groups to evaluate the effect of macroscopic structures of the renal pelvis on the SFR. In addition, intraoperative and postoperative complications were assessed using the Clavien–Dindo classification of surgical complications.^[9]^

Observation variables included the following: demographic data; the STPR on KUB analyzed by Adobe Photoshop (version 2022; Adobe Systems Incorporated, San Jose, CA); the size, position, and SCTV evaluated by NCCT; operation time (from the start of the trigger of the holmium laser equipment to laser off at the end of fURSL, excluding the time of grabbing stones with a stone extraction basket), SFR, and complications (Clavien–Dindo classification). The Ethics Review Board of the Shanxi Bethune Hospital approved this study. The study was conducted following the tenets of the Declaration of Helsinki (as revised in 2013). All patients provided written informed consent for the inclusion of their data.

### 2.3. Statistical analysis

Data were analyzed using the R 4.1.4 (Vienna Statistical Computing Foundation, Vienna, Austria) and the “survival” package. When the continuous variables did not conform to the normal distribution, they were represented by the median (interquartile range) and analyzed using the Wilcoxon rank–sum test; categorical variables are expressed as frequency and percentage (%) and calculated using the Pearson χ^2^ or Fisher exact test, depending on expected cell sizes.

Univariate and multivariate Cox proportional hazard regressions were conducted using the “survival” package to verify independent influencing factors. Furthermore, a nomogram model was established using multivariate regression. The prediction efficiency of the model was verified using the receiver operating characteristic curve and area under the curve (AUC). The bootstrap resampling method and calibration curve were used to evaluate the consistency of the model. Moreover, the net benefit of patients was analyzed using clinical decision curve analysis. Finally, the prediction model was verified in a prospective group. Differences were considered statistically significant when the *P* value was <.05.

## 3. Results

### 3.1. The baseline characteristics of patients for the study

**Figure 1.**
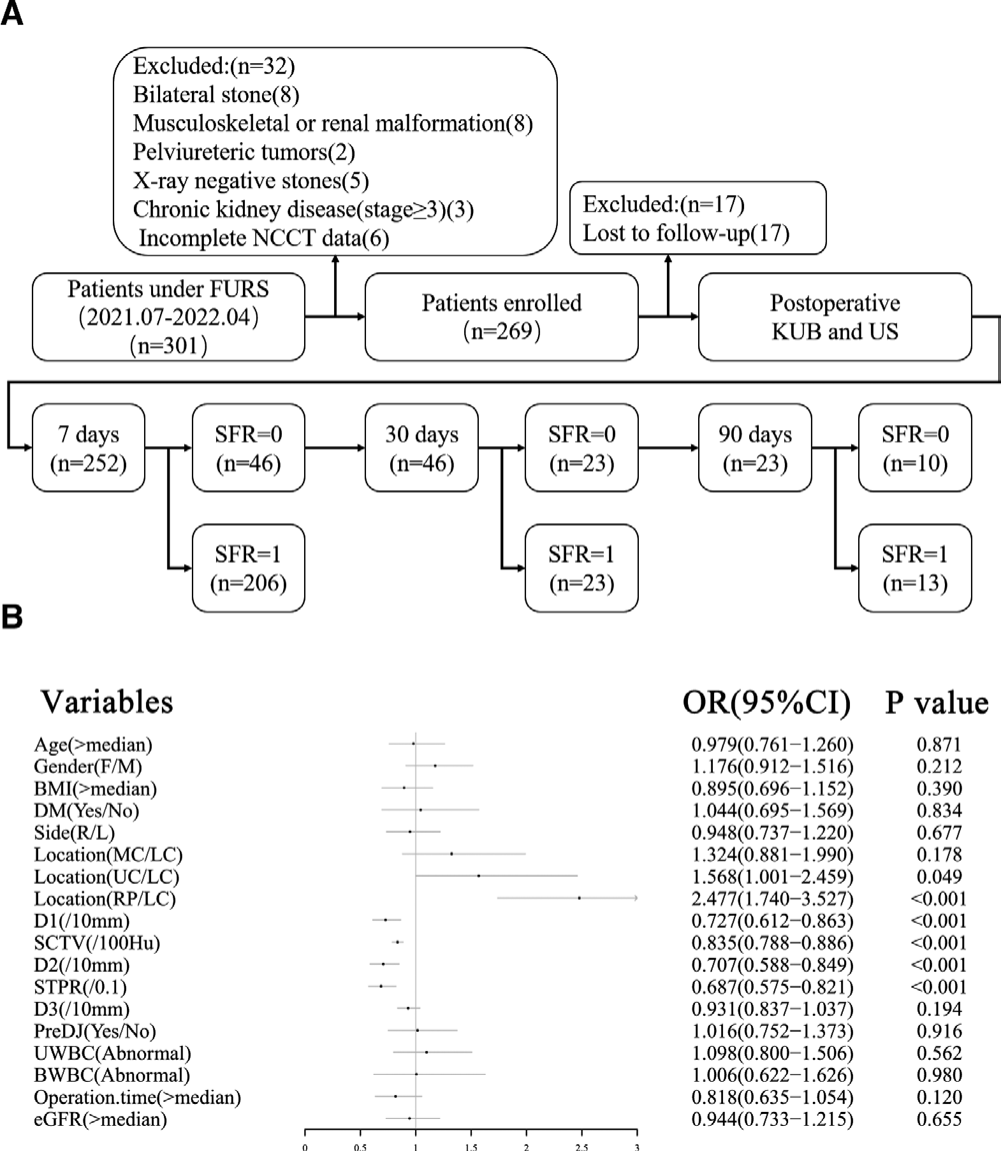
Basic information. (A) Study flowchart. (B) Forest plot of influencing factors on SFR. BWBC = white blood cell of peripheral blood, CI = confidence interval, eGFR = estimated glomerular filtration rate, MC = middle calyces of kidney, NCCT = non-contrast computed tomography, RP = renal pelvis, SFR = stone-free rate, UC = upper calyces of kidney, UWBC = white blood cell of urinalysis.

From July 2020 to April 2022, 301 patients treated with fURSL were admitted to the Shanxi Bethune Hospital. Overall, there were 8, 8, 2, 5, 3, 6, and 17 cases of bilateral renal stones, musculoskeletal or renal malformation, pelviureteric tumor, transparent stones on X-ray, severe chronic kidney disease (two of stage 3 and one of stage 4), insufficient data on radiological examination (CT examination in other hospitals before surgery), and loss to follow-up, respectively (Fig. 1A).

Finally, we enrolled 252 patients aged 23 to 67 years (median: 46.00 years [34.00–55.25]), including 141 (56.0%) males and 111 (44.0%) females; 130 (51.6%) and 122 (48.4%) patients had stones on the left and right, respectively. Among them, 58 patients underwent preoperative indwelling of double-J stents. Furthermore, 53 (21.0%), 47 (18.7%), 33 (13.1%), and 119 (47.2%) patients had stones in the lower calyce (LC), middle calyce, upper calyce (UC), and renal pelvis, respectively. The body mass index ranged from 18.76 to 44.05 kg/m^2^ (median: 24.36 kg·m^−2^ [22.38–27.77]). The diameter of the stones measured by CT (D1) ranged from 5.1 to 50.3 mm (median: 14.00 mm [10.88–19.05]), while the diameter of the stones measured by KUB (D2) ranged from 4 to 55 mm (median:13.00 mm [10.00–18.00]). The SCTV ranged from 340 to 1378 Hu (median: 831.50 Hu [713.00–929.00]). The STPR ranged from 0.80 to 1.26 (median: 1.11 [1.07–1.14]). The diameter of hydronephrosis (D3) ranged from 10 to 59 mm (median: 31.00 mm [24.00–42.00]). The operation time ranged from 20 to 196 minutes (median: 65.00 [52.00–94.25] minutes). The estimated glomerular filtration rate ranged from 39.0 to 181.0 mL/min (median: 103.0 [92.0–114.0] mL/min). There were 27, 202, 19, and 58 patients with diabetes, abnormal white blood cells on urinalysis, abnormal white blood cells in the peripheral blood, and preoperative indwelling double-J stents, respectively. In addition, when reviewed using KUB and US at 7, 30, and 90 days postoperatively, 46, 23, and 10 patients failed to discharge stones, respectively (Table 1). A significant difference was observed in the SFR at 7, 30, and 90 days postoperatively (*χ*^2^ = 28.196, *P* < .001). Complications (Clavien–Dindo classification): overall, 98 patients had complications, of which 5 patients experienced complications of Clavien–Dindo grade ≥3 (three patients underwent ESWL and URSL with nongeneral anesthesia because of residual stone obstruction after removal of the double-J stent, 1 underwent balloon dilatation of ureteral stricture under general anesthesia because of ureteral stricture, and 1 with septic shock after operation needed intensive care unit monitoring) (Table 2).

**Table 1:** Clinical characteristics of the studied group.

Variables	Total cohort (n = 252)	SFR (7 d)		*P*	SFR (30 d)		*P*	SFR (90 d)		*P*
		Yes (n = 206)	No (n = 46)		Yes (n = 229)	No (n = 23)		Yes (n = 242)	No (n = 10)	
Age	46.00 (34.00–55.25)	46.00 (34.25–55.00)	46.00 (33.25–56.00)	.964	46.00 (34.00–56.00)	46.00 (34.00–53.00)	.832	46.00 (34.00–55.75)	44.00 (37.25–51.75)	.993
Gender				.284			.348			.216
Male	141 (56.0)	112 (79.4)	29 (20.6)		126 (89.4)	15 (10.6)		133 (94.3)	8 (5.7)	
Female	111 (44.0)	94 (84.7)	17 (15.3)		103 (92.8)	8 (7.2)		109 (98.2)	2 (1.8)	
BMI	24.35 (22.38–27.77)	24.21 (22.28–27.79)	24.56 (23.16–26.77)	.514	24.33 (22.32–27.98)	24.42 (22.96–25.24)	.811	24.35 (22.37–27.86)	24.27 (22.70–24.81)	.528
DM				.821			.98			1
No	225 (89.3)	183 (81.3)	42 (18.7)		205 (91.1)	20 (8.9)		216 (96.0)	9 (4.0)	
Yes	27 (10.7)	23 (85.2)	4 (14.8)		24 (88.9)	3 (11.1)		26 (96.3)	1 (3.7)	
Side				.373			.705			.826
Left	130 (51.6)	109 (83.8)	21 (16.2)		119 (91.5)	11 (8.5)		124 (95.4)	6 (4.6)	
Right	122 (48.4)	97 (79.5)	25 (20.5)		110 (90.2)	12 (9.8)		118 (96.7)	4 (3.3)	
Location				<.001			<.001			.118
LC	53 (21.0)	31 (58.5)	22 (41.5)		41 (77.4)	12 (22.6)		48 (90.6)	5 (9.4)	
MC	47 (18.7)	33 (70.2)	14 (29.8)		41 (87.2)	6 (12.8)		45 (95.7)	2 (4.3)	
UC	33 (13.1)	26 (78.8)	7 (21.2)		30 (90.9)	3 (9.1)		32 (97.0)	1 (3.0)	
RP	119 (47.2)	116 (97.5)	3 (2.5)		117 (98.3)	2 (1.7)		117 (98.3)	2 (1.7)	
D1	14.00 (10.88–19.05)	13.00 (10.43–17.65)	18.55 (15.25–23.53)	<.001	13.40 (10.60–18.60)	21.10 (16.20–27.50)	<.001	13.90 (10.70–18.78)	22.05 (18.47–30.27)	<.001
SCTV	831.50 (713.00–929.00)	799.00 (659.50–890.75)	943.00 (903.00–1158.75)	<.001	813.00 (699.00–909.00)	1060.00 (929.00–1191.50)	<.001	825.00 (703.25–917.75)	1166.50 (1066.75–1192.75)	<.001
D2	13.00 (10.00–18.00)	12.00 (9.00–16.00)	17.50 (14.00–21.75)	<.001	13.00 (10.00–17.00)	20.00 (16.00–24.00)	<.001	13.00 (10.00–17.00)	22.50 (20.00–30.25)	<.001
STPR	1.11 (1.07–1.14)	1.11 (1.06–1.13)	1.15 (1.12–1.18)	<.001	1.11 (1.07–1.13)	1.16 (1.13–1.19)	<.001	1.11 (1.07–1.13)	1.17 (1.16–1.19)	<.001
D3	3.10 (2.40–4.20)	3.10 (2.30–4.10)	3.35 (2.52–4.40)	.148	3.10 (2.40–4.10)	3.90 (2.40–4.65)	.167	3.10 (2.40–4.20)	4.40 (3.12–4.68)	.084
PreDJ				.539			.714			.879
No	194 (77.0)	157 (80.9)	37 (19.1)		177 (91.2)	17 (8.8)		187 (96.4)	7 (3.6)	
Yes	58 (23.0)	49 (84.5)	9 (15.5)		52 (89.7)	6 (10.3)		55 (94.8)	3 (5.2)	
UWBC				.444			.607			1
Normal	50 (19.8)	39 (78.0)	11 (22.0)		44 (88.0)	6 (12.0)		48 (96.0)	2 (4.0)	
Abnormal	202 (80.2)	167 (82.7)	35 (17.3)		185 (91.6)	17 (8.4)		194 (96.0)	8 (4.0)	
BWBC				1			1			1
Normal	233 (92.5)	190 (81.5)	43 (18.5)		212 (91.0)	21 (9.0)		224 (96.1)	9 (3.9)	
Abnormal	19 (7.5)	16 (84.2)	3 (15.8)		17 (89.5)	2 (10.5)		18 (94.7)	1 (5.3)	
OT	65.00 (52.00–94.25)	64.00 (52.00–87.00)	87.00 (57.50–120.25)	.026	64.00 (52.00–92.00)	90.00 (61.00–136.00)	.025	64.00 (52.00–92.75)	108.00 (65.25–160.75)	.028
eGFR	103.00 (92.00–114.00)	103.00 (92.00–113.00)	105.50 (91.25–115.00)	.899	103.00 (92.00–113.00)	108.00 (96.50–116.50)	.262	104.00 (92.00–114.00)	99.00 (93.50–107.00)	.535

Continuous variables were represented by the median (interquartile range) and analyzed using the Wilcoxon rank–sum test; categorical variables are expressed as frequency and percentage (%) and calculated using the Pearson *χ*^2^ or Fisher exact test.

BMI = body mass index, BWBC = white blood cell of peripheral blood, D = days, D1 = diameter of stone measured by CT, D2 = diameter of stone measured by KUB, D3 = diameter of hydronephrosis, DM = diabetes mellitus, KUB = kidney-ureter-bladder abdominal radiography, LC = lower calyces of kidney, MC = middle calyces of kidney, OT = operation time, PreDJ = preoperative indwelling of double-J stent, RP = renal pelvis, SCTV = CT value of stone, SFR = stone-free rate, STPR = stone and transverse process pixel ratio, UC = upper calyces of kidney, UWBC = white blood cell of urinalysis.

**Table 2:** Classification of complications.

Complications	I	II	IIIa	IIIb	IVa	Summation
Pain (back, abdomen, or flank)	44					44
Fever	24					24
Hematuria	18					18
Stone residues (3 mo)	7		3			10
Ureteral stricture				1		1
Septic shock					1	1
Total	93	0	3	1	1	98

### 3.2. Univariate Cox proportional hazard regression

**Figure 2.**
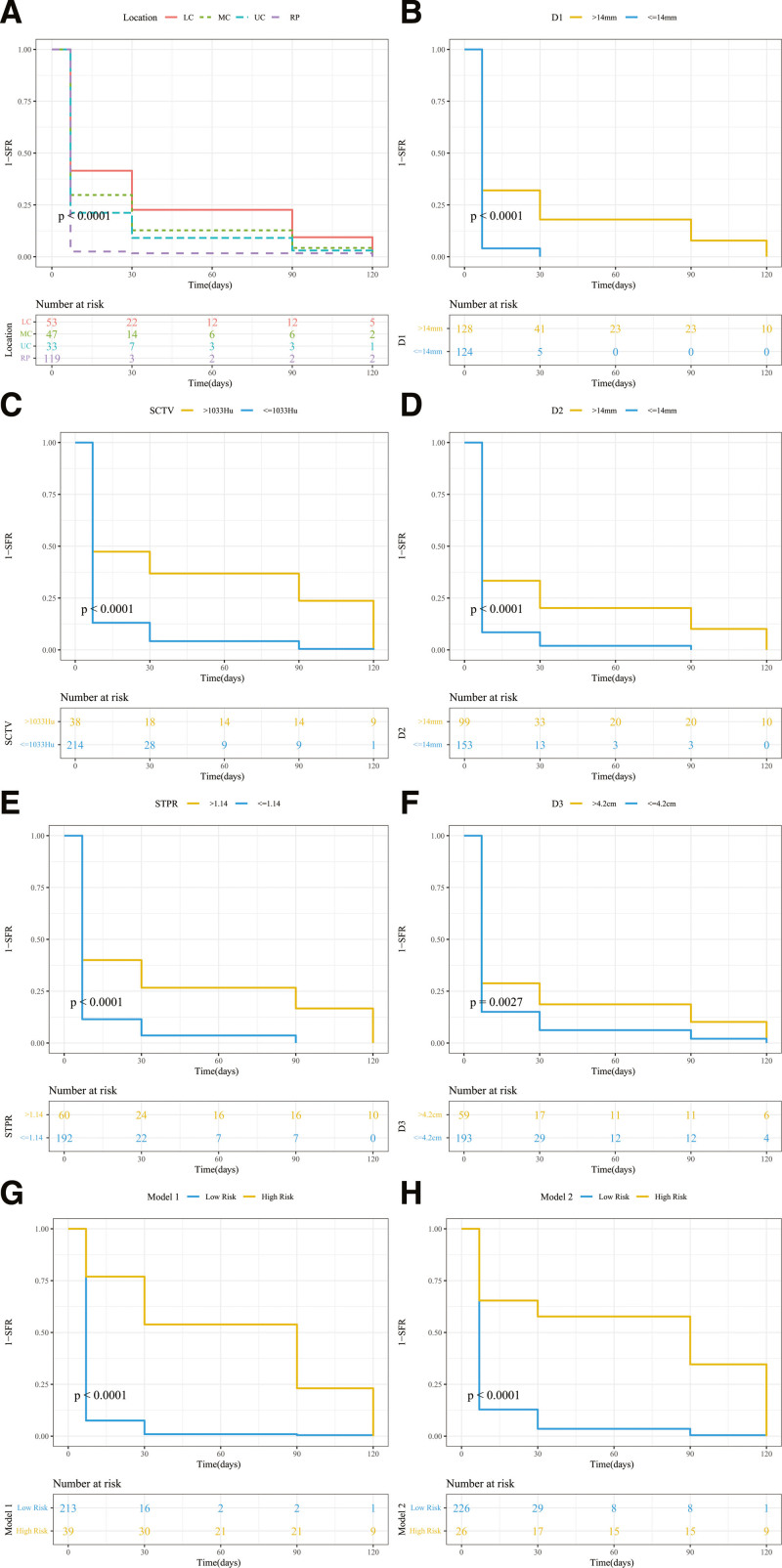
Kaplan–Meier survival curves. (A) Location. (B) D1. (C) SCTV. (D) D2. (E) STPR. (F) D3. (G) Model 1. (H) Model 2. CT = computed tomography, SCTV = CT value of stone, STPR = stone/transverse process pixel ratio.

Univariate Cox proportional hazards regression results are shown in Figure 1B, implying that SFR was correlated with location (upper calyce compared with LC) (hazard ratio [HR], 1.568; 95% confidence interval [CI], 1.001–2.459, *P* = .049), location (renal pelvis compared with LC) (HR, 2.477; 95% CI, 1.740–3.527, *P* < .001), D1 (HR, 0.969; 95% CI, 0.952–0.985, *P* < .001), SCTV (HR, 0.998; 95% CI, 0.998–0.999, *P* < .001), D2 (HR, 0.966; 95% CI, 0.948–0.983, *P* < .001), STPR (HR, 0.023; 95% CI, 0.004–0.139, *P* < .001), operation time (HR, 0.996; 95% CI, 0.992–0.999, *P* = .008) (Fig. 2A–D).

Prediction models of the SFR were constructed using multivariate regression (Table 3). Because there was collinearity between SCTV and STPR (Pearson correlation = 0.453, *P *< .001), the following 2 models were constructed: model 1: the included influencing factors are D1, location, and SCTV; model 2: the included influencing factors are D2, location, and STPR (Fig. 2E and F).

**Table 3:** Multivariate regression of SFR.

Variables	Model 1 (SCTV)			Model 2 (STPR)		
	HR	95% CI	*P*	HR	95% CI	*P*
Location			.050			.034
LC	1		–	1		–
MC	1.239	0.823–1.864	.305	1.246	0.826–1.879	.295
UC	1.260	0.801–1.981	.317	1.302	0.829–2.044	.252
RP	1.634	1.142–2.339	.007	1.689	1.176–2.427	.005
D1/D2	0.984	0.970–0.999	.043	0.984	0.968–0.999	.045
SCTV/STPR	0.999	0.998–0.999	.002	0.087	0.013–0.596	.013

Prediction models of the SFR were constructed using multivariate regression.

D1 = diameter of stone measured by CT, D2 = diameter of stone measured by KUB, KUB = kidney-ureter-bladder abdominal radiography, LC = lower calyces of kidney, MC = middle calyces of kidney, RP = renal pelvis, SCTV = CT value of stone, SFR = stone-free rate, STPR = stone and transverse process pixel ratio, UC = upper calyces of kidney.

### 3.3. Nomogram

**Figure 3.**
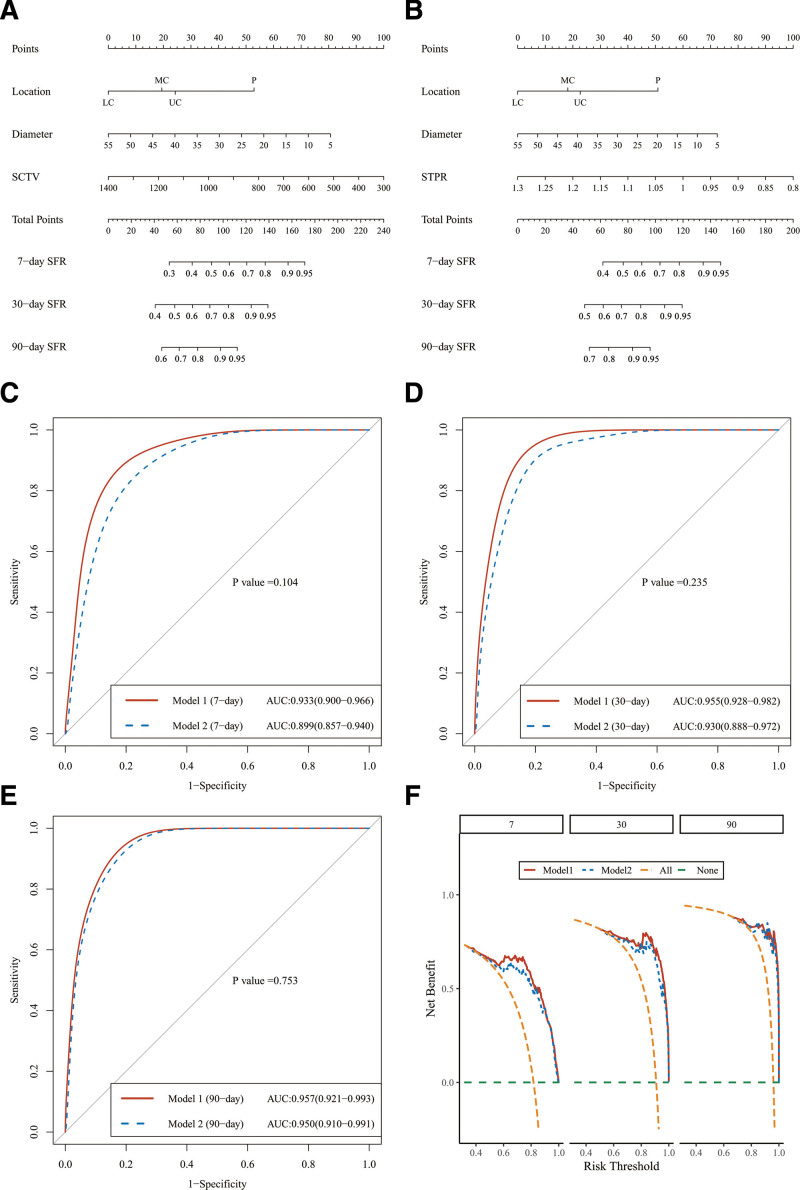
Validation and performance of the nomogram. (A) Nomogram (model 1). (B) Nomogram (model 2). (C) ROC curve at 7 d. (D) ROC curve at 30 d. (E) ROC curve at 90 d. (F) DCA curves. DCA = decision curve analysis, ROC = receiver operating characteristic.

The results of the multivariate regression are shown in the nomogram (Fig. 3A and B). Points for each parameter are assigned corresponding values from the “points” axis, and the sum of the points is plotted on the “total points” axis to calculate the SFR. Probability is the value at a vertical line from the corresponding total points. For example, in model 2, a patient had a stone with a D2 of 20 mm in the LC, the STPR was 1.10, and corresponding scores of each index were 0, 50, and 40, respectively, with a total score of 90. The predicted SFRs were 60%, 78%, and 92% for 7, 30, and 90 days after the operation, respectively.

### 3.4. Verification and performance of the nomogram

The receiver operating characteristic curve was plotted according to the nomograms. No significant difference was observed between models 1 and 2 (*P* = .498). After 1000 resamplings and internal self-validation, the C-index of models 1 and 2 was 0.924 (95% CI: 0.893–0.955) and 0.895 (95% CI: 0.858–0.932), respectively. The models revealed that the SFR predicted by the nomogram was consistent with that of the actual situation. In model 1, the AUC was 0.933 (95% CI: 0.900–0.966), 0.955 (95% CI:0.928–0.982), and 0.957 (95% CI: 0.921–0.993) at 7, 30, and 90 days, respectively. Furthermore, in model 2, the AUC was 00.899 (95% CI: 0.857–0.940), 0.930 (95% CI: 0.888–0.972), and 0.950 (95% CI: 0.910–0.991) at 7, 30, and 90 days, respectively, indicating that the nomogram has a highly accurate predictive ability (Fig. 3C and E).

Decision curve analysis based on model 1 and model 2, can be seen that the models yielded a net benefit for patients of approximately 50%, 65%, and 75% at 7, 30, and 90 days, respectively (Fig. 3F).

Furthermore, based on the predicted risk levels from models 1 and 2 and utilizing the optimal cutoff points for grouping, the SFR of the low-risk group significantly outperforms that of the high-risk group (Fig. 2G–H).

## 4. Discussion

Currently, the AUA and EAU guidelines recommend fURSL as the first-line treatment method for renal stones whose diameter is <20 mm. PCNL is recommended as the first-line treatment for others. However, with the improvement of fURSL, including technique and equipment, a growing number of patients choose fURSL rather than PCNL because of its risk and contraindications. Fayad et al^[10]^ believed that treating renal stones with diameters between 20 and 30 mm should consider the benefits and disease risks for the patient. The primary purpose of surgical treatment of renal stones is to ensure fewer postoperative complications and a higher SFR, which requires establishing a scoring system capable of predicting SFR and selecting the most appropriate type of operation that can be admitted by both doctors and patients.^[4]^ Over the past few years, a series of scoring systems have been established to evaluate SFR after fURSL. The common point is that stone load and location are significantly correlated with SFR^[5,11–13]^; meanwhile, whether stone density affects SFR is controversial.

In this study, fURSL was used to treat renal stones. In addition, the size, location, and density of stones were determined to be independent factors influencing fURSL.^[5,11–13]^ Large stone volumes, those located in the LC, and acute pelvic infundibular angle affect SFR after fURSL. This finding is consistent with the results of other studies. Correlating with SFR, stone density is an essential parameter for predicting the SFR of URSL.^[14,15]^ Yamashita et al^[16]^ analyzed the mean density of renal stones using 3-dimensional NCCT and confirmed that it could predict the prognosis of renal stones treated with URSL. The higher the density of the stones, the more complex the operation. Furthermore, Gucuk et al^[14]^ compared fURSL with PCNL in treating renal stones <20 mm and concluded that the success rate was similar; however, stone density significantly affected SFR. NCCT is an important method that is commonly used to evaluate stone density. Generally, it is believed that stones with a CT value >1000 Hu are considered to have a hard texture. Although US and magnetic resonance imaging are radiation-free, they cannot evaluate stone density. In this study, fURSL was used to treat renal stones. The results of this study showed that, in addition to stone load and location, stone density was an independent factor affecting SFR after fURSL. However, it is also a concern that high doses of CT radiation may negatively affect patients’ long-term health.

Some scholars have analyzed the KUB of stones and compared the stone density with that of the adjacent vertebra’s 12-costal cartilage or transverse process. They found that the effect of higher-density stones treated with ESWL was unsatisfactory.^[17,18]^ However, this is only a subjective evaluation.

Inspired by the reasons above, this study proposed a critical parameter, STPR, which is obtained by analyzing KUB and measuring the average pixel value of stones and adjacent vertebral transverse processes. Statistical analysis revealed that STPR was significantly correlated with SCTV and was an independent influencing factor affecting the SFR after fURSL. This study constructed a new CT-based scoring system and a prediction model using KUB for the first time. Simultaneously, SFR prediction models were constructed respectively at days 7, 30, and 90 after fURSL. We compared the prediction model based on KUB with that of the NCCT. The results showed no significant difference in the prediction efficiency between the 2 models (*P* = .498), and the prediction effect of both models was satisfactory. Therefore, this effect is satisfactory. The advantage of establishing the KUB prediction model is that it is simple, convenient, and beneficial for doctor-patient communication because the result of NCCT is too complex to understand for patients. Simultaneously, KUB’s advantages are that it is intuitive and easy to understand, and the postoperative follow-up is convenient. Therefore, KUB can be an alternative to NCCT because it reduces the dose of radiation received by patients to protect their health and is helpful for surgeons to elaborate on the effect of fURSL preoperatively, which is convenient for the choice of individual operation plan. In addition, economically, the cost of intravenous urography is approximately 4 times higher than that of KUB, and the cost of NCCT is 4 times higher than that of intravenous urography, which reduces the financial burden on patients to a certain extent and saves medical resources.^[19]^ However, KUB is easily affected by pneumatosis and intestinal contents. Liu et al^[20]^ found that intestinal preparation influenced the evaluation of stone detection and density characteristics. The detection rate of stones with a diameter of >5 mm diagnosed by KUB was 56.6% and 73.5% before and after intestinal preparation, respectively. Therefore, model 2 cannot replace model 1. However, model 2 can be used in some special cases. For example, STPR can be used instead of SCTV to evaluate stone density if obtaining the related parameter from CT proves to be challenging or impractical.

Furthermore, some studies have shown that the fURSL operation time is positively correlated with the size of stones; the longer the operation time, the greater the probability of complications.^[21]^ The operator’s experience is correlated with the operation time and the likelihood of ureteric perforation but does not affect the SFR treated by fURSL.^[22]^ Disposable equipment for fURS could shorten the operation time and is a practical treatment choice for larger stones.^[23]^ A study by Komeya et al^[24]^ showed that when the gap between the ureteroscope and ureteral sheath was more than 6 mm, it was more beneficial to fURSL, and the SFR was higher. The fURSL procedure was performed at our center by experienced urologists at our center using disposable equipment and avoided the personnel and equipment influencing SFR.

However, this study had certain limitations. This study lacks multicenter clinical trials and requires external and prospective validation from a larger cohort.

In conclusion, stones’ location, size, and density are independent factors influencing the SFR during fURSL. To our knowledge, this is the first report of nomograms for predicting SFR after fURSL based on CT and KUB. The KUB-based scoring system sometimes proved to be just as effective as the CT-based one. This study has important clinical significance as its findings may be used to guide optimal treatment strategy selection.

## Author contributions

**Data curation:** Bin Yang, Shiwei Sun.

**Project administration:** Bin Yang, Jinyao Wang, Xiaofeng Yang.

**Writing**—**original draft:** Bin Yang, Shiwei Sun, Jinyao Wang, Shuqin Liu, Yangbing Wei.

**Formal analysis:** Shiwei Sun, Shuqin Liu.

**Writing**—**review & editing:** Jingyu Wang, Xiaofeng Yang.
